# Manganese-Doped Calcium Silicate Nanowire Composite Hydrogels for Melanoma Treatment and Wound Healing

**DOI:** 10.34133/2021/9780943

**Published:** 2021-05-07

**Authors:** Zhongcao Wu, Hui Zhuang, Bing Ma, Yin Xiao, Bahattin Koc, Yufang Zhu, Chengtie Wu

**Affiliations:** ^1^State Key Laboratory of High Performance Ceramics and Superfine Microstructure, Shanghai Institute of Ceramics, Chinese Academy of Sciences, Shanghai 200050, China; ^2^Center of Materials Science and Optoelectronics Engineering, University of Chinese Academy of Sciences, Beijing 100049, China; ^3^School of Materials Science and Engineering, Shanghai University, Shanghai 200444, China; ^4^Institute of Health and Biomedical Innovation, Queensland University of Technology, 7 Brisbane 4059, Australia; ^5^Integrated Manufacturing Technologies Research and Application Center, Sabanci University, Tuzla 34956 Istanbul, Turkey; ^6^SUNUM Nanotechnology Research Center, Sabanci University, Tuzla 34956 Istanbul, Turkey; ^7^Faculty of Engineering and Natural Sciences, Sabanci University, Tuzla 34956 Istanbul, Turkey

## Abstract

Melanoma is a serious malignant skin tumor. Effectively eliminating melanoma and healing after-surgical wounds are always challenges in clinical studies. To address these problems, we propose manganese-doped calcium silicate nanowire-incorporated alginate hydrogels (named MCSA hydrogels) for *in situ* photothermal ablation of melanoma followed by the wound healing process. The proposed MCSA hydrogel had controllable gelation properties, reasonable strength, and excellent bioactivity due to the incorporated calcium silicate nanowires as the *in situ* cross-linking agents and bioactive components. The doping of manganese into calcium silicate nanowires gave them excellent photothermal effects for eradicating melanoma effectively under near infrared (NIR) irradiation. Moreover, the synergistic effect of manganese and silicon in the MCSA hydrogel effectively promotes migration and proliferation of vascular endothelial cells and promotes angiogenesis. Hence, such bifunctional bioactive hydrogels could achieve combined functions of photothermal therapy and wound healing, showing great promise for melanoma therapy and tissue regeneration.

## 1. Introduction

Melanoma is the most lethal form of skin cancer, which is mainly caused by excessive ultraviolet radiation [1]. Since it is prone to lymph node and blood metastasis, melanoma has a high lethality rate and poor prognosis [2]. Nowadays, surgical resection combined with chemotherapy/radiotherapy is one of the commonly used therapeutic approaches for melanoma therapy [3]. However, incomplete surgical resection usually causes recurrence, and the refractory wounds left by the surgery are hard to self-heal, even prone to secondary injury due to infection. Consequently, there are pressing needs to develop multifunctional biomaterials with simultaneous tumor-killing and skin tissue regeneration capability for desirable therapeutic efficacy.

Photothermal therapy (PTT) has emerged as a highly efficient strategy for tumor therapy due to its high selectivity, minimal invasiveness, and no systemic effect compared with traditional chemotherapy/radiotherapy [4, 5]. The melanoma occurs in superficial anatomical location, where the laser beam used in PTT can penetrate [6]. Thus, PTT possesses superiority in curing melanoma. According to previous studies, it has been demonstrated that the PTT showed remarkable therapeutic performance for melanoma therapy [7–9]. However, the photothermal agents still suffer from some drawbacks in the melanoma therapy, such as limited bioactivity and acute inflammation, which limit further clinical applications of PTT.

In order to eliminate residual tumor cells and repair wounds caused by surgical excision, a new therapeutic platform with good biocompatibility and high efficiency of photothermal conversion and wound repair effect is needed. Silicate bioceramics offer a great prospect in the field of tissue engineering including skin [10], bone [11], and cartilage [12] regeneration. SiO_4_^4-^ released from silicate bioceramics can stimulate vascularization and further promote wound healing [13–16]. Moreover, the manganese (Mn) element plays a vital role in extracellular matrix synthesis and shows great potential in bone tissue regeneration [17, 18]. However, the effect of manganese on wound healing has not been investigated in the literature. Besides, the incorporation of transition elements (Fe, Co, and Mn) could endow silicate bioceramics with excellent photothermal performance [17]. Consequently, it is reasonable to speculate that Mn-doped silicate biomaterials can possess both tumor-killing and wound healing capabilities, which offer high potential for melanoma therapy and wound healing.

The bioceramic powders still retain notable challenges in clinical applications when directly applied for wound healing, such as a high pH value at wound sites, nonuniform distribution, and potential cytotoxicity caused by excessive metal ions [19–21]. To solve these problems, an appropriate wound dressing biomaterial could be used to load the bioceramic powders. Sodium alginate (SA) is a kind of natural linear anionic polysaccharide from algae, which is widely used in wound dressing and other tissue engineering fields [22–24]. Divalent metallic ions, such as Ca^2+^, Zn^2+^, and Mn^2+^, can chelate with sodium alginate solution, resulting in the cross-linking of molecular segments that forms a network structure and transforms the solution into a gel [25]. However, the reaction is too fast to form homogenous hydrogels if exogenous divalent metallic ions are introduced into the sodium alginate solution directly [23]. It has been demonstrated that silicate bioceramics could slowly release divalent metallic ions under mild acid conditions [23, 26]. Consequently, it is reasonable to assume that the incorporation of Mn-doped calcium silicate (MCS) into SA hydrogels can result in a controllable gelation process to obtain homogenous hydrogels.

**Scheme 1:**
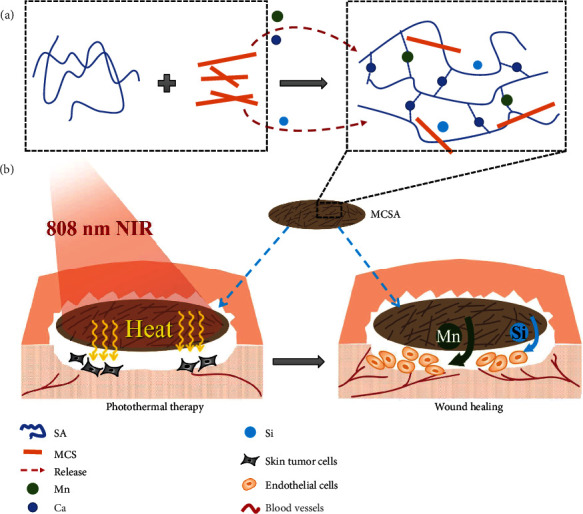
Schematic illustration of the fabrication and application of MCSA composite hydrogels. (a) MCSA composite hydrogels were prepared by incorporating Mn-doped calcium silicate nanowires into hydrogels, which possessed controllable gelation process with the release of Mn and Ca ions from MCS. (b) Photothermal therapy of melanoma and enhanced wound healing based on the MCSA composite hydrogels.

In this study, a bifunctional composite hydrogel was prepared by incorporating Mn-doped calcium silicate nanowires into a sodium alginate hydrogel for postoperative treatment of malignant melanoma (Scheme 1). Calcium and manganese ions released from Mn-doped calcium silicate nanowires effectively controlled the gelation process of hydrogels. With the introduction of Mn, the composite hydrogels were endowed with excellent photothermal properties, resulting in effective photothermal therapeutic efficacy both *in vitro* and *in vivo*. Furthermore, the proposed composite hydrogel could promote the wound healing in both tumor-bearing and diabetic mice. This kind of bifunctional composite hydrogels provide a promising strategy for simultaneously killing tumor cells and repairing skin tissues after surgical excision of melanoma.

## 2. Results and Discussion

### 2.1. Characterization of CS and MCS Nanowires

**Figure 1:**
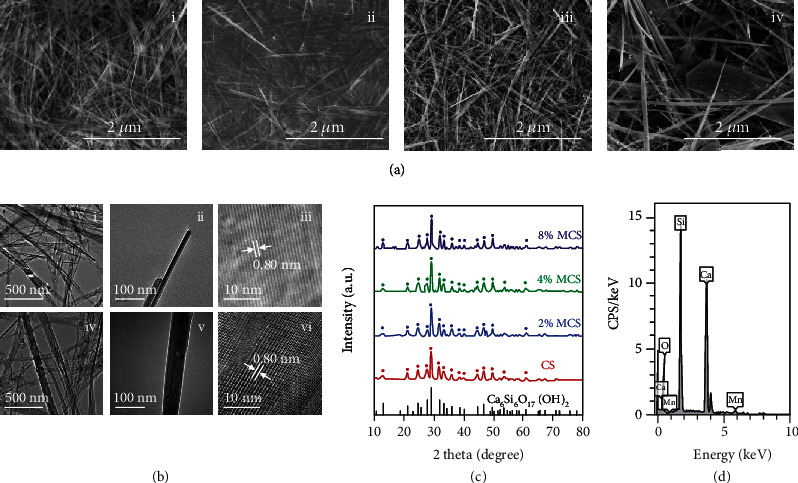
Characterization of CS and MCS nanowires. (a) Scanning electron microscope (SEM) images of (i) CS nanowires, (ii) 2% MCS nanowires, (iii) 4% MCS nanowires, and (iv) 8% MCS nanowires. (b) TEM images of (i–ii) CS nanowires and (iv–v) 4% MCS nanowires; HRTEM images of (iii) CS nanowires and (vi) 4% MCS nanowires. (c) XRD patterns of CS, 2% MCS, 4% MCS, and 8% MCS nanowires. (d) EDS analysis of 4% MCS nanowires.

High-quality calcium silicate (CS) and manganese-doped calcium silicate (MCS) nanowires were synthesized by the reaction of Ca(NO_3_)_2_ and Na_2_SiO_3_ with or without MnCl_2_*via* a hydrothermal treatment at 200°C. The morphology and structure of CS and MCS nanowires (Figures 1(a) and 1(b)) were characterized by scanning electron microscopy (SEM) and transmission electron microscopy (TEM). The length of nanowires for each sample was at the microscale, and the diameter ranged from 10 nm to 100 nm (Figure 1(a)). Under the same hydrothermal conditions (200°C, 24 h), the morphology of nanowires was affected by the Mn content. Pure CS nanowires had a long vertical length (≥10 *μ*m) and small diameter (≤20 nm). With the increase of Mn doping, the nanowires changed from soft wires to needles. The diameter of the 8% MCS nanowires was more than 100 nm, and some flack shape particles were mixed in the nanowires. The radius of Mn^2+^ is 81 pm, and that of Ca^2+^ is 114 pm. A smaller ion radius enabled Mn^2+^ to enter the vacancy of silicate or replace Ca^2+^ to form doping. As shown in high-resolution transmission electron microscope (HRTEM) images (Figure 1(b)), CS nanowires showed high crystallinity (Figure 1(b), iii), while MCS nanowires possessed higher defect content, including a large number of atomic dislocations, stacking faults, and slips (Figure 1(b), vi), making MCS nanowires' crystal structure more complex than that of CS nanowires. According to the X-ray diffraction (XRD) patterns (Figure 1(c)), the peaks of CS nanowires were well indexed into the xonotlite phase (PDF#03-0568). In addition, the introduction of Mn showed little effect on the phase of nanowires. The energy-dispersive spectroscope (EDS) detected the Mn element in MCS nanowires (Figure 1(d)).

### 2.2. Characterization of CA and Composite Hydrogels

**Figure 2:**
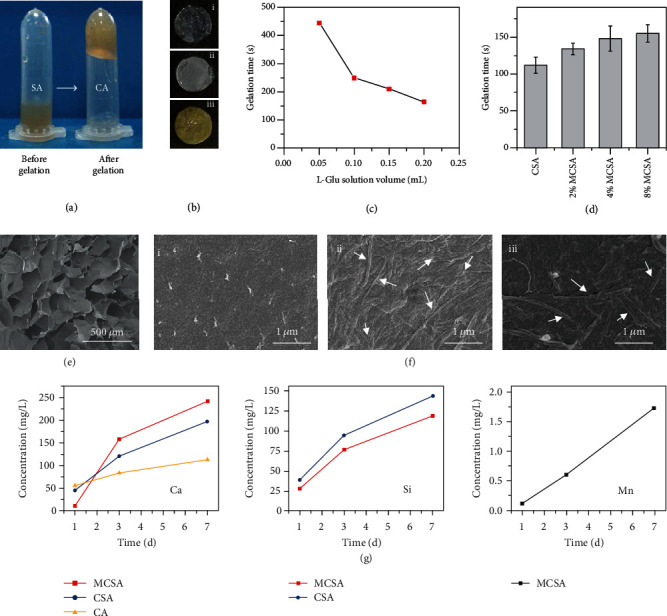
Characterization of composite hydrogels containing CS and MCS nanowires. (a) Photographs of the hydrogel before and after gelation. (b) Photographs of the hydrogels: (i) CA, (ii) CSA, and (iii) 4% MCSA. (c) The relationship between the gelation time and the L-Glu solution volume. (d) The relationship between the gelation time and the Mn content of MCSA. (e) Porous structure of the cross-section of CA hydrogel. (f) Scanning electron microscope (SEM) images of the surface of calcium alginate hydrogels with CS or MCS after lyophilization: (i) CA, (ii) CSA, and (iii) 4% MCSA. The arrows indicate the nanowires. (g) Concentration of Ca, Si, and Mn ions released from CA, CSA, and MCSA hydrogels (1, 3, and 7 days).

To utilize the developed MCS nanowires for tumor treatment and wound repair, a biocompatible alginate hydrogel was chosen to be the matrix material. Sodium alginate (SA) can dissolve in aqueous solution and quickly change to calcium alginate (CA) hydrogel when cross-linked by Ca^2+^ in the solution. In this study, L-glutamic acid (L-Glu) was used as a gelling agent to construct alginate hydrogels with CS or MCS incorporation. CS or MCS nanowires can accelerate the release of Ca^2+^ with the help of the L-Glu gelling agent and thereby promote the gelation of sodium alginate. When the hydrogel stagnated at the bottom of the centrifuge tube, it was considered to be polymerized totally (Figure 2(a)). Herein, L-Glu as a gelling agent could control the release of Ca^2+^ from CS or MCS nanowires, and thereby, the gelation time could be controlled within several minutes when the L-Glu solution was added dropwise (Figure 2(c)). Mn^2+^ can also facilitate the cross-linking of alginate, but the cross-linking process is slower than that of Ca^2+^ [25]. Therefore, for 2% MCSA, 4% MCSA, and 8% MCSA hydrogels, the gelation was gradually slowed due to the increase of Mn content (Figure 2(d)). With such a mechanism, we can regulate the gelation rate of the composite hydrogel in a certain range. The hydrogels were shaped to sheets with a Teflon mold (*d* = 10 *μ*m, thickness = 0.8 mm) for the subsequent characterization and evaluation. The CA hydrogel was colorless and transparent, the CS-alginate (CSA) hydrogel appeared white, and the MCS-alginate (MCSA) hydrogel appeared light brown. The color of hydrogels was influenced with the incorporation of CS and MCS nanowires, and it further deepened with the increase of the Mn content in MCS nanowires (Figure 2(b)). SEM observation of the hydrogels after lyophilization showed a porous structure with a pore size of 200 *μ*m for CA hydrogels (Figure 2(e)), which can absorb and hold large amounts of water and aqueous solutions. CS and MCS nanowires were then dispersed homogeneously in hydrogels as shown in Figure 2(f). There were no differences in the morphologies and sizes of the CS and MCS nanowires compared with the nanowires dispersed in anhydrous ethanol, which indicated that the fabrication process of hydrogels did not destroy the CS and MCS nanowires. The gels were immersed into an aqueous Tris-HCl buffer solution (200 *μ*L/mL, pH = 7.4, 37°C), and the ion concentration of Ca, Si, and Mn was measured at day 1, day 3, and day 7. Ca^2+^ and Mn^2+^ exhibited a uniform release rate. After the doping of Mn, the nanowires exhibited a trend with quicker release of Ca and slower release of Si. Due to the smaller ionic radius of Mn^2+^ compared with that of Ca^2+^, the addition of Mn might result in more vacancies and defects in the crystal unit and consequently caused the release of Ca ions from MCSA to be higher than that from CSA [27]. In addition, the binding energy of Ca-Si (245 eV) in xonotlite was lower than that of Mn-Si (642 eV) that led to more stable performance of MCSA with lower release of Si ions. The Ca^2+^ with a greater release rate played a major role in the gelation process, and Mn^2+^ could adjust the gelation performance by changing the doping amount. The previous studies have demonstrated that Si ions were proven to have favorable effects on cell proliferation and differentiation and tissue regeneration [28, 29], and Mn ions were proven to contribute to the synthesis of the cytoplasmic matrix [17, 18]. Hence, the MCSA hydrogel could potentially promote wound healing.

### 2.3. Photothermal Performance of Nanowires and Composite Hydrogels

**Figure 3:**
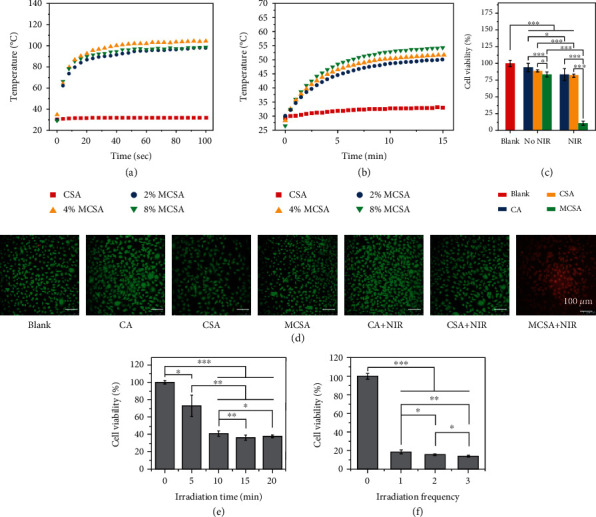
Evaluation of photothermal effect of nanowires and composite hydrogels. (a) Photothermal heating curves of the CS and MCS nanowires. (b) Photothermal heating curves of the CSA and MCSA hydrogels. (c) *In vitro* B16F10 cell viability after being treated by different hydrogels with/without photothermal therapy and (d) the corresponding live/dead staining observed by confocal microscopy. (e) *In vitro* B16F10 cell viability after being treated by the MCSA hydrogel with different NIR irradiation times. (f) *In vitro* B16F10 cell viability after being treated by the MCSA hydrogel with different NIR irradiation frequencies.

As shown in Figure 3(a), with an 808 nm irradiation at a power density of 0.4 W/cm^2^, the temperature of CS nanowires did not increase, but the temperature of the MCS nanowires with different doping amounts (2% MCS, 4% MCS, and 8% MCS) increases rapidly to above 90°C within 30 seconds. This proves that the Mn doping effectively improves the photothermal effect of CS nanowires. Correspondingly, photothermal heating curves of the hydrogels exhibited similar trends compared with the nanowires. The CSA hydrogel showed little temperature increase after NIR irradiation at 0.4 W/cm^2^, while the temperatures of the MCSA hydrogels (2% MCSA, 4% MCSA, and 8% MCSA) increased rapidly from 30°C to above 45°C within 5 minutes after NIR irradiation at 0.4 W/cm^2^ (Figure 3(b)). Local photothermal temperature above 45°C can kill tumors effectively [4, 30]; thus, the MCSA hydrogels can effectively kill tumor cells with an appropriate photothermal treatment. To explore the photothermal effect of MCSA hydrogels *in vitro*, the murine skin melanoma cells (B16F10) were seeded in 24-well plates containing hydrogels. The cells of the NIR irradiation groups were irradiated by an 808 nm laser (2.60 W/cm^2^) for 15 minutes while no special treatment was exerted to the other groups, then all the cells were costained with Calcein-AM and Ethidium Homodimer-1 to distinguish live (green) and dead (red) cells by the observation with a confocal microscope (Figure 3(d)). It was observed that there were no residual live tumor cells in the MCSA+NIR group, and no significant live cell decrease occurred in the other groups. The cell viability of each group was determined by a CCK-8 (Cell Counting Kit-8, Beyotime) assay to quantitatively measure the photothermal capability of the hydrogels. As shown in Figure 3(c), the viability of cells incubated with MCSA hydrogels decreased to 10.6% after NIR irradiation, while the cell viability in other groups was each above 80%. In order to achieve the optimal photothermal therapy effect, a series of evaluations were also carried out with various times and frequencies of NIR irradiation, and the cell viability was also determined by CCK-8 assay. Under a 5-minute irradiation, the cell survival rate began to decrease significantly but still maintained at 72.92%, which could not effectively kill the cells. When the irradiation time was over 10 minutes, the cell viability decreased to a low level (Figure 3(e)). After a 15-minute irradiation, the cell viability decreased to 14.84%, the second and the third irradiation further reduced the cell viability to 11.97% and 10.52%, respectively, but would not effectively improve the treatment efficiency (Figure 3(f)). These results concluded that the MCSA hydrogels could efficiently kill skin melanoma cells via photothermal effect with an 808 nm laser irradiation once and longer than 10 minutes.

### 2.4. Antitumor Therapeutic Efficacy *In Vivo* Based on Composite Hydrogels

**Figure 4:**
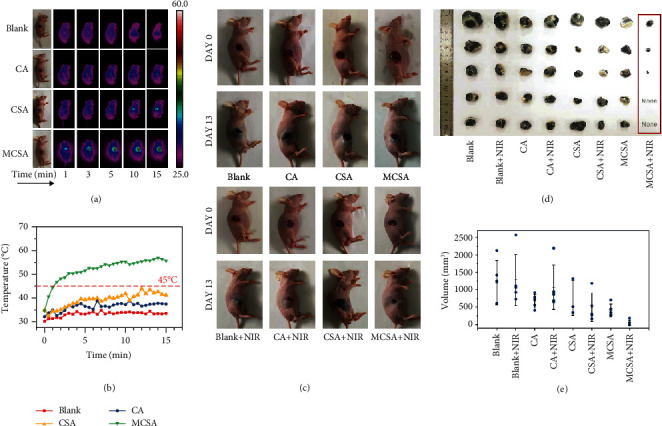
Antitumor efficiency of the MCSA hydrogels. (a) Infrared thermal images of B16F10 tumor-bearing mice treated with blank, CA, CSA, and MCSA hydrogels under 808 nm laser irradiation (1.8 W/cm^2^, 15 min). (b) Photothermal heating curves of the mouse skin surface temperature of the MCSA group corresponding to the irradiation time. (c) Representative photographs of B16F10 tumor-bearing mice after various treatments on day 0 and day 13. (d, e) Tumor photographs and tumor volume statistics from B16F10 tumor-bearing mice after 14-day treatment.

*In vivo* photothermal therapy was realized by the mouse subcutaneous melanoma model. A round full-thickness skin wound (diameter = 10 mm) was created above each tumor site when the tumor volume reached about 50 mm^3^. The wounds were covered by the CA, CSA, and MCSA hydrogels (200 *μ*L) and then exposed to 808 nm laser irradiation (1.80 W/cm^2^, 15 min) for 4 consecutive days. The wounds covered by the CA, CSA, and MCSA hydrogels in the absence of laser irradiation were used as control groups. For all the groups, the real-time thermal images were monitored by an infrared imaging device during the irradiation; simultaneously, the temperature changes were recorded (Figure 4(a)). The photothermal heating curve of the MCSA hydrogel showed that the real-time temperature of the therapeutic site rose above 45°C after 1-minute irradiation and finally reached to 55°C, indicating the possibility to kill tumor cells effectively. The photothermal heating curves of the other groups did not reach to 45°C. From the representative photographs of B16F10 tumor-bearing mice after various treatments on day 0 and day 13, it is shown that the uninhibited tumors make wounds more difficult to contract, which is necessary for wound healing (Figure 4(b)). After 4 days of photothermal treatment, the tumor growth of the MCSA group was suppressed, while the tumor volume in the other groups was boosted in an uncontrollable manner. Tumor photographs and tumor volume scatter plot could directly reflect the photothermal therapeutic effect of the hydrogels (Figures 4(c) and 4(d)). The skin melanoma tumor growth was significantly inhibited by the laser-irradiated MCSA hydrogels, and the wounds gradually healed without obvious tumor recurrence within 14 days.

**Figure 5:**
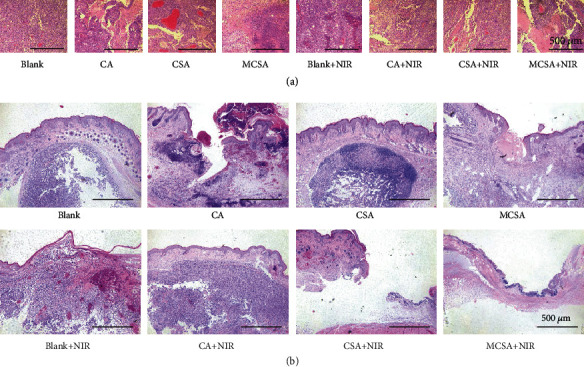
Histological analysis of tumors and healing skins. (a) H&E staining of sectioned tumors for 14 days after various treatments. (b) H&E staining of sectioned healing skins for 14 days after various treatments.

The removed tumors at day 13 were stained with hematoxylin and eosin (H&E). Most of tumor cells in the MCSA+NIR group were apoptotic and exfoliated; the residual tumor tissue structure was loose and normal cells began to grow, while the tumor sections of the other groups showed closely arranged melanoma cells (Figure 5(a)). New skin formation from the healed wound area by skin resection was also stained with H&E for histological analysis. In the MCSA+NIR group, the newly formed tissue highly resembles the normal skin with aligned tissue architectures and regular capillaries, while there were irregular cavities, plentiful residual tumor cells, and disordered tumor vascularization observed in the other groups (Figure 5(b)). According to the results above, the conjecture could be confirmed that tumor cells were eradicated by the photothermal therapy in the early treatment stage, and then skin defects induced by skin tumors were stimulated and repaired with the MCSA hydrogels.

### 2.5. *In Vitro* Biocompatibility of Composite Hydrogels

**Figure 6:**
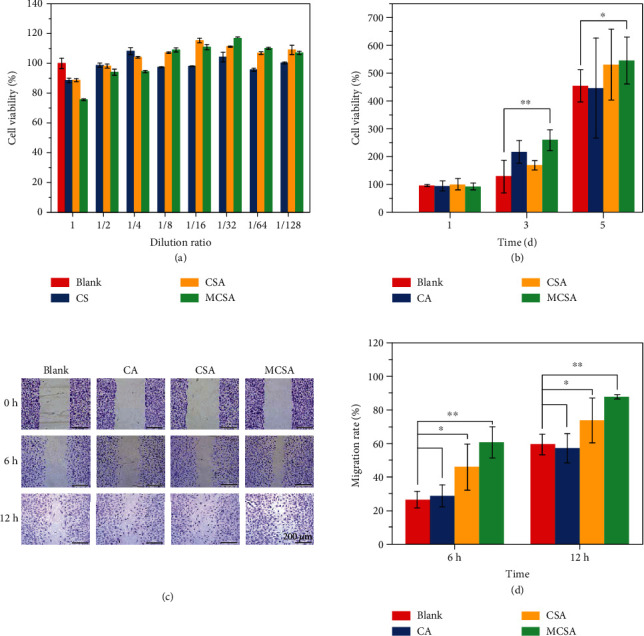
*In vitro* bioactivity of composite hydrogel extracts. (a) *In vitro* HUVEC cell viability with various hydrogel extract treatments (CA, CSA, and MCSA hydrogels; the dilution rate is 1, 1/2, 1/4, 1/8, 1/16, and 1/32; 24 h). (b) *In vitro* HUVEC cell viability with 1/32 dilution of extract (CA, CSA, and MCSA hydrogels and cultured for 1, 3, and 5 days). (c) *In vitro* scratch assay of HUVECs cultured with the MCSA, CSA, and CA hydrogel extracts. (d) Migration rate of HUVECs.

Wound healing is a complex regulatory process involving many cell types such as macrophages, keratinocytes, endothelial cells, and fibroblasts interacting with each other [31, 32]. Their production, migration, proliferation, and differentiation influence the occurrence of inflammatory reaction and tissue regeneration, which ultimately induce wound healing. Endothelial cells (EC) exist in the vascular tissue, and plasma synthesize and secrete a variety of endothelial growth factors and thereby control substances in and out of blood vessels [33]. Therefore, their growth marks the formation of blood vessels, which is of great significance for tissue regeneration. In this study, human umbilical vein endothelial cells (HUVECs) were employed to evaluate the tissue regeneration ability of the MCSA hydrogels. Extracts of various hydrogels were diluted with an endothelial cell growth medium (ECM total, dilution ratio = 1, 1/2, 1/4, 1/8, 1/16, 1/32, 1/64, and 1/128), and the cell viability was determined by a CCK-8 assay after being cultured for 24 h to evaluate the cytotoxicity of the hydrogel extracts (CA, CSA, and MCSA). At a high concentration, too much metal ions and alkalinity beyond the range hindered the growth of the cells and inhibited the proliferation, but when the dilution ratio was lower than 1/8, the inhibition turned to a promotion effect. The performance of the CSA hydrogel was similar to that of the MCSA hydrogels. There was no significant difference between the CA hydrogel and control groups (Figure 6(a)). We carried out cell proliferation and migration experiments at the dilution ratio of 1/32. HUVECs were cultured with the extracts of hydrogels for 5 days. The day 3 and day 5 results showed that the cell viability of the MCSA group was significantly higher than that of the other groups (Figure 6(b)), indicating that the Mn ions released from the MCSA hydrogel could promote HUVEC proliferation. In addition, the *in vitro* scratch test was used to investigate the effect of hydrogels on cell migration. Figure 6(c) showed the migration of cells in the scratch at 0 h, 6 h, and 12 h, and the migration rate was calculated (Figure 6(d)). It was found that cell migration could be promoted by both CSA and MCSA hydrogel groups, and the MCSA group was better than the CSA group. However, the CA group did not show any significant difference on cell migration compared with the control group. It can be speculated that the Si and Mn ions released from the MCSA hydrogel could play a synergistic role in promoting wound healing.

### 2.6. Effect of Composite Hydrogels on Wound Healing *In Vivo*

**Figure 7:**
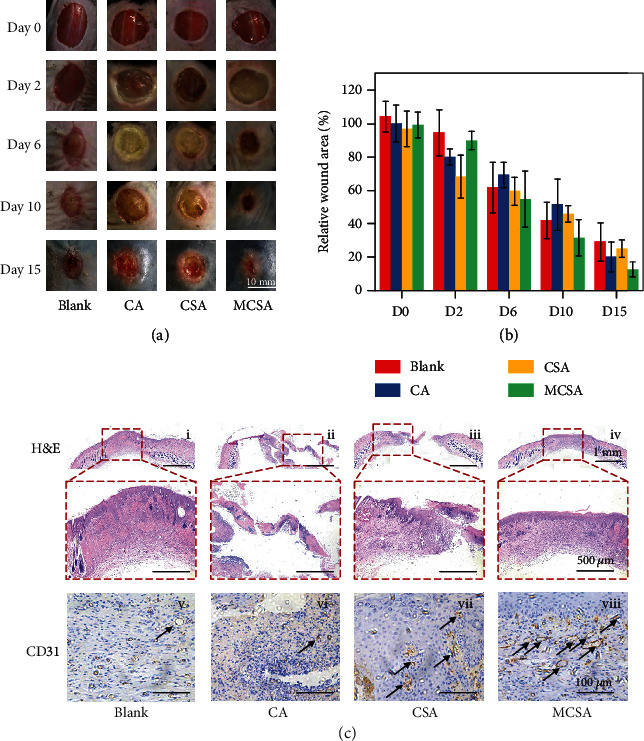
Diabetic wound healing performance by composite hydrogels. (a) Gross photographs of murine skin wounds on days 0, 2, 6, 10, and 15 after various treatments. (b) The relative wound area of mice treated by various hydrogels on days 0, 2, 6, 10, and 15. (c) H&E and CD31 staining of sectioned skin for 16 days after various treatments (black arrows: blood vessels).

*In vivo* chronic skin wound healing effect of the developed hydrogel was evaluated in a diabetic mouse model. A round full-thickness skin wound (diameter = 10 mm) was created on the back of diabetic mice after shaving. The wounds were treated with various hydrogels for 16 days as diabetic wounds without hydrogel treatment were used as the control.  Figure 7(a) shows that the healing process of the MCSA group was better than the other groups, which was better than the other groups. Quantitative statistics of all the chronic wounds revealed that the MCSA group had the lowest relative wound area at day 15 (Figure 7(b)). The results of tissue staining showed that the MCSA group not only healed faster, but also had a higher density of blood vessels, hair follicles, glands, and other skin appendages (Figure 7(c)), which confirmed that the composite hydrogel could protect the microenvironment of the wound as well as effectively promote the maturity of the new skin tissue. Hydrogels can absorb the exudate from the wound surface to accelerate the wound healing process [13]. Simultaneously, the released Si and Mn ions also play an important role in the cell proliferation and promoting wound healing. Overall, the MCSA hydrogels could serve as an effective and promising material for promoting cutaneous tissue regeneration.

## 3. Conclusions

In summary, we have successfully synthesized Mn-doped calcium silicate (MCS) nanowires with a photothermal effect, which was incorporated in an alginate hydrogel to fabricate a bifunctional bioactive hydrogel (MCSA) integrating photothermal therapy and wound healing processes. The MCSA hydrogels possessed a controllable gelation process under the trigger of a mild acid microenvironment to slowly release divalent metallic ions. The existence of Mn in calcium silicate nanowires provided an excellent photothermal property with NIR laser irradiation and thereby a photothermal therapeutic outcome against skin melanoma tumor *in vitro* and *in vivo*. Meanwhile, the composite hydrogel, which contained bioactive ions (Mn^2+^ and SiO_4_^4-^), significantly accelerated the wound healing process. The results showed that the developed bifunctional MCSA hydrogels would be an excellent candidate for integrative melanoma photothermal therapy and wound healing processes.

## 4. Materials and Methods

### 4.1. Synthesis of CS and MCS Nanowires

The CS and MCS nanowires were both synthesized via a hydrothermal treatment. For the synthesis of MCS nanowires, calcium nitrate tetrahydrate (Ca(NO_3_)_2_·4H_2_O, Aladdin, 99%) and sodium metasilicate nonahydrate (Na_2_SiO_3_·9H_2_O, Sinopharm Chemical Reagent Co., Ltd., AR) were dissolved in deionized water (0.4 mol/L). After dissolving completely, both solutions were mixed and stirred till they turned into a white suspension. Then, manganese chloride tetrahydrate (MnCl_2_·4H_2_O, Collins, 99.99%) was added to the mixed solution of Ca(NO_3_)_2_·4H_2_O and Na_2_SiO_3_·9H_2_O with the Mn/Si atomic ratio of 2%, 4%, and 8%. Subsequently, the suspension was transferred into Teflon-lined stainless-steel autoclaves and treated at 200°C for 24 h. After hydrothermal treatment, the products were washed with water and anhydrous ethanol three times, and MCS nanowires were obtained and stored in anhydrous ethanol at 4°C.

For the synthesis of CS nanowires, the process was the same as that for MCS nanowires without the addition of manganese chloride tetrahydrate, and CS nanowires were also stored in anhydrous ethanol. Anhydrous ethanol should be washed out before further experiments.

### 4.2. Preparation of CA, CSA, and MCSA Hydrogels

Sodium alginate (SA, low viscosity, Alfa Aesar) solution (3.0 wt. %) was obtained by dissolving SA powder in deionized water. Subsequently, the SA solution was pipetted into 48-well plates (200 *μ*L per well). CS and MCS nanowires were added to the SA solution by an inorganic/organic mass ratio of 1/2 (dry weight). The SA solution was polymerized by the addition of the cross-linking agent L(+)-glutamic acid (L-Glu, Aladdin, 99%) to help release Ca^2+^ from CS and MCS. As a control, the CA hydrogel was prepared by adding 100 *μ*L calcium chloride (CaCl_2_, Aladdin, 96.0%) solution (2.5 wt. %) per well in the SA solution. The hydrogels were preserved in CaCl_2_ solution in the shape of round sheets (10 mm in diameter, 0.8 mm in thickness).

### 4.3. Characterization

The chemical composition of various nanowires was determined by X-ray diffraction (XRD, Rigaku, Japan) and scanning electron microscopy (SEM, SU8200, Hitachi, Tokyo, Japan) equipped with an energy-dispersive spectrometer (EDS). The morphologies of nanowires were observed by scanning electron microscopy (SEM, 4800, Hitachi, Tokyo, Japan) and transmission electron microscopy (TEM, JEM-2100F, JEOL, Japan). HRTEM images were taken by transmission electron microscopy. The concentrations of Ca, Si, and Mn ions released from hydrogels were measured by inductively coupled plasma-atomic emission spectrometry (ICP-AES, Vista AX, Varian, Palo Alto, CA, US). The light-induced temperature changes, and thermal images of hydrogels were monitored using an infrared imaging device (PM100D, Thorlabs GmbH, Munich, Germany). Fluorescence images were recorded by confocal laser scanning microscopy (CLSM, TCS SP8, Leica, Germany).

### 4.4. Photothermal Performance of MCS Nanowires and MCSA Hydrogels

MCS and CS nanowires stored in anhydrous ethanol were dried in 60°C for 24 h before testing photothermal performance. The real-time thermal images were monitored by an infrared imaging device during the irradiation; simultaneously, the temperature changes were recorded. Nanowires with different Mn contents (CS, 2% MCS, 4% MCS, and 8% MCS) were tested with an 808 nm laser at various laser power densities (0.1 W/cm^2^, 0.2 W/cm^2^, 0.4 W/cm^2^, and 0.6 W/cm^2^), respectively, for 100 seconds. The photothermal performance of MCSA hydrogels was tested in 48-well culture plates. The real-time thermal images and temperature changes were recorded with the same methods. Hydrogels with different concentrations of CS and MCS nanowires (CSA, 2% MCSA, 4% MCSA, and 8% MCSA) were tested with an 808 nm laser at various laser power densities (0.4 W/cm^2^, 0.6 W/cm^2^, and 1.0 W/cm^2^), respectively, for 15 min. The effects of the inorganic component content (16.7%, 25%, 33.3%, and 50%) and SA concentration (1.5, 3.0 g/100 g H_2_O) on the photothermal performance of the composite hydrogels were also investigated with 4% MCS concentration under the same experimental condition.

### 4.5. Photothermal Therapy *In Vitro*

The murine skin melanoma cells (B16F10) were cultured in Roswell Park Memorial Institute 1640 Medium (RPMI 1640, Gibco) with 10% fetal bovine serum (FBS, Gibco) and 1% penicillin-streptomycin (PS) in a humidified incubator (5% CO_2_, 37°C). The photothermal therapeutic effect *in vitro* of 4% MCSA hydrogel was evaluated in 24-well plates. Each well contains 600 *μ*L cell culture medium and equivalent CA, CSA, and MCSA hydrogels (200 *μ*L). B16F10 cells were seeded onto slides in plates (8 × 10^4^ cells/well). After being cultured for 24 h in a humidified incubator, the slides with cells were transferred into the 24-well plates and cultured for 15 min. For the irradiation group, the cells were irradiated by an 808 nm laser (2.60 W/cm^2^) while no special treatment was given to other groups. Cells were costained with Calcein-AM and Ethidium Homodimer-1 to distinguish live (green) and dead (red) states and observed with a confocal laser scanning microscope. Meanwhile, the cell survival rate was calculated quantitatively, and the cell viability of each group was determined by a CCK-8 (Cell Counting Kit-8, Beyotime) assay. CCK-8 solution was diluted with 10% proportion by cell complete medium and then added in the plates (300 *μ*L per well). After incubation for 1.5 h, the absorbance of the solution at 450 nm was measured by a microplate reader. The absorbance of the diluted CCK-8 solution before incubation was denoted as *A*_0_ and the absorbance after incubation in the blank group and other groups was denoted as *A*_Blank_ and *A*_Sample_, respectively. The cell viability was calculated according to the following equation: Cell viability (%) = (*A*_Sample_ − *A*_0_)/(*A*_Blank_ − *A*_0_)∗100%. The effects of irradiation time (0, 5, 10, 15, and 20 min) and irradiation frequency (0, 1, 2, and 3 times) on cell viability were also determined by CCK-8 assay.

### 4.6. *In Vivo* Photothermal Therapy and Tumor-Induced Wound Healing

All animal studies were conducted in accordance with protocols approved by the Institutional Animal Care and Use Committee of Nanjing First Hospital, Nanjing Medical University. The subcutaneous melanoma model was established with Balb/c mice (male, 7-8 weeks old) by injecting 5 × 10^5^ B16F10 cells subcutaneously into the right flank of each mouse. Experimental mice were divided into 8 groups randomly, which were (1) blank, (2) blank+NIR, (3) CA, (4) CA+NIR, (5) CSA, (6) CSA+NIR, (7) MCSA, and (8) MCSA+NIR. 4% MCSA hydrogels were used in the *in vivo* experiments. A round full-thickness skin wound (diameter = 10 mm) was created directly above each tumor site when the tumor volume reached about 50 mm^3^ (tumor diameter: 4-5 mm). Subsequently, the corresponding hydrogels were applied to the exposed tumor site through the whole experiment process, and NIR irradiation (808 nm laser, 1.80 W/cm^2^, 15 min) was exerted to groups (2), (4), (6), and (8) for the first 4 days. The surface temperature was monitored in real time. Photographs were taken, and the tumor volumes were record every other day. After 14 days of treatment, all the mice were sacrificed, and the tumors were measured and photographed. Then, the tumors and skin tissues from the wound bed were excised and were fixed with a 4% (*v*/*v*) paraformaldehyde solution and embedded in paraffin and stained with hematoxylin and eosin (H&E). The tumor and skin specimens were observed and photographed with an optical microscope.

### 4.7. Bioactivity of the Composite Hydrogels *In Vitro*

Human umbilical vein endothelial cells (HUVECs) were cultured in a specified endothelial medium (ECM, ScienCell) in a humidified incubator (5% CO_2_, 37°C). The extract of the hydrogel was prepared according to the standard (ISO 10993-5). Cytotoxicity of the hydrogel extracts was tested in 48-well plates. HUVECs were seeded into plates (4 × 10^5^ cells/well, 500 *μ*L medium) and incubated for 12 h, then the culture medium was replaced by a 500 *μ*L extract of the CA, CSA, or MCSA hydrogels (dilution ratio = 1, 1/2, 1/4, 1/8, 1/16, 1/32, 1/64, and 1/128). Cells without treatment of hydrogel extracts were used as a control. The cell viability was determined by the CCK-8 assay after being cultured for 24 h.

For the cell proliferation assay, HUVECs were incubated in 96-well plates (600 cells/well) for 24 h. Each well was added with a 100 *μ*L extract (CA, CSA, and MCSA; dilution ratio = 1/32), and the cell viability was determined by the CCK-8 assay after being cultured for 1, 3, and 5 days.

For evaluation of cell migration behavior, an *in vitro* scratch assay was carried out. HUVECs (1 × 10^6^ cells/well, 1 mL medium) were seeded into 12-well plates and incubated for 12 h, followed by serum-free starvation to reset the cell cycle. A vertical scratch was created in the confluent cell monolayer using a 200 *μ*L pipet tip, then the cells were incubated in a 1 mL extract, and cells without treatment of hydrogel extracts were used as a control. At 0 h, 6 h, and 12 h, cells were fixed with paraformaldehyde (4%), stained in crystal violet hydrate solution (0.1%), and photographed by microscopy (DMI3000, Leica, Germany). Finally, the migration rate was calculated according to the photographs by ImageJ software.

### 4.8. *In Vivo* Chronic Skin Wound Healing

The diabetic wound model was established with C57BL/6 mice (male, 7-8 weeks old). The mice were treated with intraperitoneal injection of streptozocin (STZ, Sigma-Aldrich, 500 mg/kg, 0.1 M in citrate buffer solution, pH = 4.5). When the blood glucose level exceeded 20 mmol/L, the mice were considered diabetic. Experimental mice were divided into 4 groups randomly, which were (1) blank, (2) CA, (3) CSA, and (4) MCSA. A round full-thickness skin wound (diameter = 10 mm) was created at the center of each mouse's back after removing the hair in the back area. Subsequently, the corresponding hydrogels were applied to the wound area. Photographs of the wound area were taken every few days, and the area of the wound was measured by ImageJ software. After being treated for 16 days, all the mice were sacrificed. The skin tissues from the wound bed were excised and fixed with 4% (*v*/*v*) paraformaldehyde solution and embedded in paraffin, then stained with hematoxylin and eosin (H&E) and CD31. The skin specimens were observed and photographed with an optical microscope.

### 4.9. Statistical Analysis

All data in this study were analyzed and expressed as mean ± standard deviation by one-way analysis. The significant difference was considered when *p* < 0.05 (∗), *p* < 0.01 (∗∗), and *p* < 0.001 (∗∗∗).

## Data Availability

The data that support the findings of this study are available from the corresponding author upon reasonable request.
